# Supplementary material for: Predicting visual function by interpreting a neuronal wiring diagram
Source: Nature. 2024 Oct 2;634(8032):113–23. doi: 10.1038/s41586-024-07953-5 (PMC11446822; doi:10.1038/s41586-024-07953-5)
Supplement: Supplementary file 6 — CRF and ERF predictions for individual TmY4 and TmY9 cells. Analogous to Supplementary Data 3, but for TmY target types. Shown are the top four monosynaptic pathways, the strongest pathway passing through each of the top ten intermediary types (ranking from Extended Data Fig. 7), and the trisynaptic pathway Tm1–TmY–Dm3–TmY (see the section entitled Prediction of spatial normalization). [file 41586_2024_7953_MOESM6_ESM.zip › DataS4/TmY9qΓèÑ/720575940626703460.pdf]

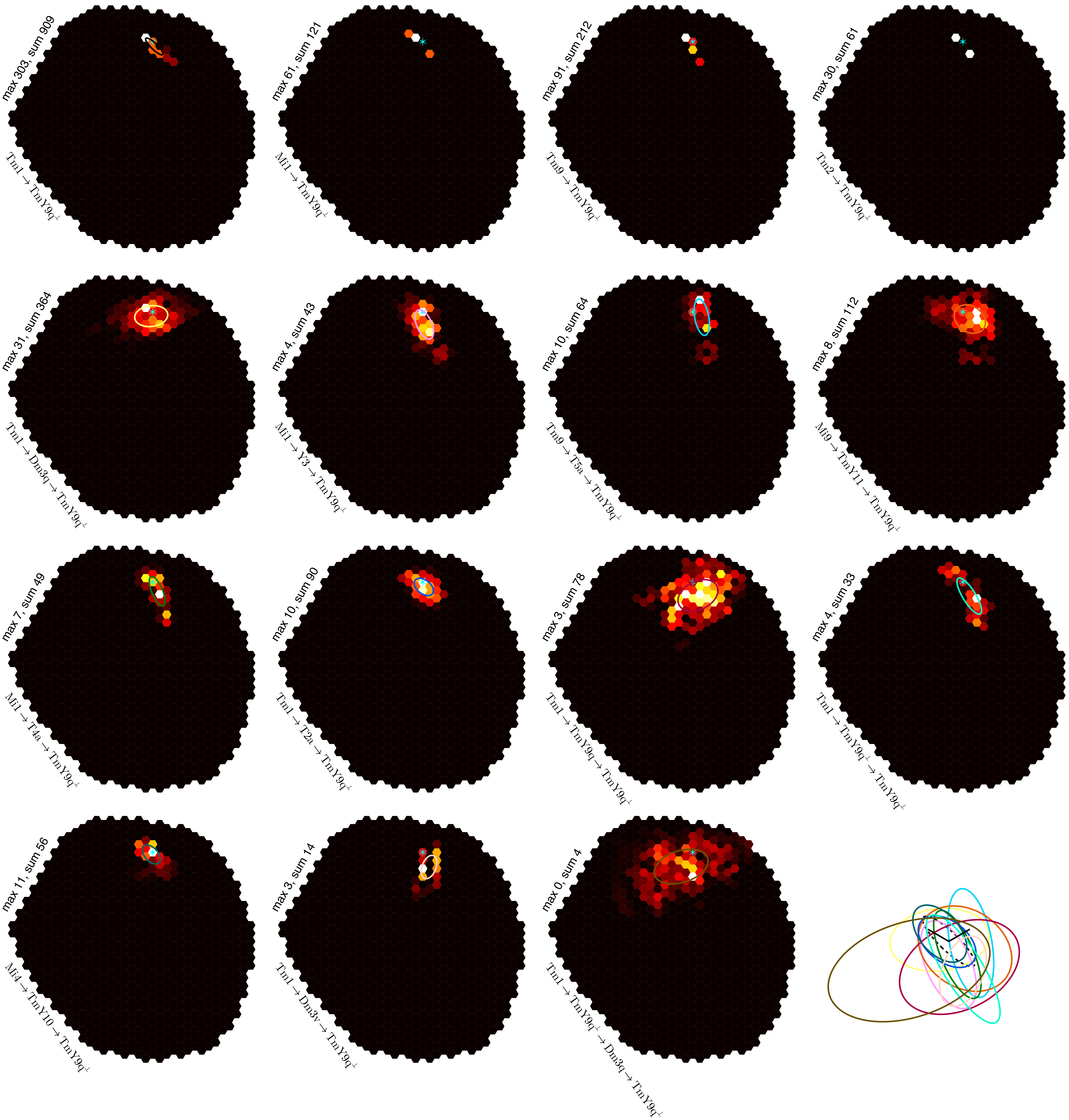
max 303, sum 909
Tm1 → TmY9q⊥
max 61, sum 121
Mi1 → TmY9q⊥
max 91, sum 212
Tm9 → TmY9q⊥
max 30, sum 61
Tm2 → TmY9q⊥
max 31, sum 364
Tm1 → Dm3q → TmY9q⊥
max 4, sum 43
Mi1 → Y3 → TmY9q⊥
max 10, sum 64
Tm9 → T5a → TmY9q⊥
max 8, sum 112
Mi9 → TmY11 → TmY9q⊥
max 7, sum 49
Mi1 → T4a → TmY9q⊥
max 10, sum 90
Tm1 → T2a → TmY9q⊥
max 3, sum 78
Tm1 → TmY9q → TmY9q⊥
max 4, sum 33
Tm1 → TmY9q⊥ → TmY9q⊥
max 11, sum 56
Mi4 → TmY10 → TmY9q⊥
max 3, sum 14
Tm1 → Dm3v → TmY9q⊥
max 0, sum 4
Tm1 → TmY9q⊥ → Dm3q → TmY9q⊥
